# Cupric citrate supplementation improves growth performance, nutrient utilization, antioxidant capacity, and intestinal microbiota of broilers

**DOI:** 10.5713/ab.24.0382

**Published:** 2024-10-25

**Authors:** Xuezhuang Wu, Yahao Zhou, Zhentao Lu, Yunting Zhang, Tietao Zhang, Qingkui Jiang

**Affiliations:** 1 College of Animal Science, Anhui Science and Technology University, Anhui 233100, China; 2 Institute of Special Animal and Plant Sciences, Chinese Academy of Agricultural Sciences, Changchun 130112, China; 3 Public Health Research Institute, New Jersey Medical School, Rutgers Biomedical and Health Sciences, Rutgers, The State University of New Jersey, Newark, NJ 07103, USA

**Keywords:** Antioxidant Defenses, Broiler, Copper Supplementation, Cupric Citrate, Intestinal Microbiota

## Abstract

**Objective:**

This study aimed to examine the impact of cupric citrate on broilers and compare it with the copper sulfate groups and a control group.

**Methods:**

A total of 360 1-day-old Ross 308 broilers were randomly assigned into 5 groups, each with 6 replicates of 12 broilers per treatment. The control group was fed a basal diet without any copper supplementation. In contrast, the other groups received basal diets supplemented with either 50 mg/kg (CS-50) or 100 mg/kg (CS-100) of copper in the form of copper sulfate, or 50 mg/kg (CC-50) or 100 mg/kg (CC-100) of copper in the form of cupric citrate, for a period of 42 days.

**Results:**

The results showed that copper supplementation affected the average daily gain (ADG) from day 1 to 21 (p = 0.026) and day 1 to 42 (p = 0.025) in a source-dependent manner. Copper source also influenced the energy digestibility (p = 0.004), with the CC-100 being the most effective treatment. Notably, birds in the CC-100 groups had significantly reduced concentrations of *Escherichia coli* (p<0.05) in the cecum, and the *Lactobacillus* in the ileum, compared to the control group. Dietary copper supplementation also increased the pH in the duodenum (p<0.05) irrespective of the sources and levels. In addition, the source of copper affected the activities of ceruloplasmin (p = 0.014) and Cu-Zn superoxide dismutase (p = 0.025) in the serum, with the CC-100 group showing the highest levels of both enzymes.

**Conclusion:**

Copper supplementation generally improves the growth, nutrient utilization, intestinal microflora, gastrointestinal pH, and antioxidant defences of broilers. Moreover, cupric citrate is as effective as copper sulfate even at equal or lower concentrations.

## INTRODUCTION

Copper, a trace element widely distributed in organisms, plays diverse roles in various biological processes, including energy metabolism, metabolic reactions, and antioxidant functions. It also has a broad bactericidal spectrum, effective against bacteria, molds and fungi [1]. In 1945, it was reported that adding high copper (100 to 250 mg/kg) to feed can improve the growth performance of piglets [2]. Subsequent studies confirmed that feeding high copper can enhance the average daily gain (ADG), average daily feed intake (ADFI) and feed conversion ratio (F/G) of animals [3,4]. Moreover, copper supplementation has been shown to reduce the pH value of intestinal chyme and improve the activities of proteinase and phospholipase [5,6].

Although copper plays a pivotal role in livestock farming, the source of this essential element warrants careful consideration. The origin of copper influences not only the cost of feed, but also its stability, bioavailability, and ultimately, its effectiveness in farming practices. Copper sulfate, an inorganic salt, has been consistently employed in poultry production for almost 100 years [7]. Despite being cost-effective, copper sulfate has high water solubility due to weak ionic bonds. It generates free copper ions during the feed manufacturing process, feed consumption, and manure disposal, leading to reduced bioavailability and environmental toxicity. Therefore, there has been a concerted effort by us and others to find alternatives to traditional copper sulfate used in the poultry industry [8–11].

Citric acid is an important bioactive compound and a chelating ligand with strong coordination ability [12]. Citric acid chelates have the advantages of high bioavailability and a lower price compared to other organic acid chelates, making them more aligned with market demand [13,14]. Cupric citrate is a chemical compound that combines copper with citric acid, resulting in a bioavailable form of copper that can be easily absorbed by living organisms. Cupric citrate has been argued to reduce nutrients destruction and has high biological potency. Adding 60 mg/kg cupric citrate to piglet diet improves growth performance, reduces copper residue in the body, enhances antioxidant function [15]. Supplementing piglet diets with 20 mg/kg cupric citrate has been shown to reduce the diarrhea rate, likely by increasing serum lysozyme concentration and levels of the antimicrobial peptides protegrins 1, as evidenced by upregulated mRNA levels of NPG1 in the bone marrow of weaning piglet [16]. Previous studies have demonstrated that cupric citrate can promote growth in chickens and reduce Cu accumulations in litter [17,18] Due to its potential in feed industry, cupric citrate has been newly approved as a novel feed additive by the Ministry of Agriculture and Rural Affairs of China in 2019. However, studies on dietary cupric citrate supplementation in broilers are outdated and may not reflect current understanding. The systematic effects of cupric citrate on poultry are not well investigated. To this end, we studied the effects of dietary cupric citrate supplementation on the growth performance, nutrient utilization, intestinal microbiota and antioxidant capacity of Ross 308 broilers. This study aims to provide a basis for the scientific application of cupric citrate in broiler production.

## MATERIALS AND METHODS

### Animal care

Ethics committee approval was received from Anhui Science and Technology University Ethics Committee with decision number 2021012 on 01/03/2021. The experiment was conducted in accordance with guidelines approved by the Animal Health and Care Committee of Anhui Science and Technology University. The protocol for the present experiment was approved by the Animal Care Committee of the Anhui Science and Technology University (2021012).

### Animals, diets, and management

Three hundred and sixty 1-day-old healthy broiler chicks (Ross 308) were allotted to 5 groups with 6 replicates in each group and 12 broilers in each replicate. Dietary treatments were as follows: (1) basal diet without supplemental copper (control); (2) basal diet+50 mg copper/kg dry matter (DM) in the form of copper sulfate (CS-50); (3) basal diet+100 mg copper/kg DM in the form of copper sulfate (CS-100); (4) basal diet+50 mg copper/kg DM in the form of cupric citrate (CC-50) and (5) basal diet+100 mg copper/kg DM in the form of cupric citrate (CC-100). The experiment lasted for 42 days, and diets were formulated in two stages (1 to 21 days old and 22 to 42 days old). Ingredients and chemical composition of the basal diets are presented in Table 1. The final copper content of the diets is presented in Table 2.

The single-layer cage (200 cm long×100 cm wide×40 cm high, with concrete floors of 0.167 m^2^/bird) was adopted in the experiment. The chicken house and related appliances were cleaned and disinfected before the experiment. The temperature of the chicken house was controlled and maintained using an industrial temperature control system starting three days prior to the experiment. Diets and water (less than 0.01 mg/L copper by analysis) were available *ad libitum*.

### Sample collection

On the d 1, d 21 and d 42, the weight was measured (stop feeding for 12 hours before weighing and drink freely), and the feed intake was recorded at the same time. ADG, ADFI and the F/G were calculated based on the recorded data.

On day 42, 60 birds (2 chicks per replicate) were randomly selected and weighed after feed deprivation for 12 h. Blood samples were collected from the wing vein and centrifuged at 3,500×g for 10 min. Serum was separated and stored at −80°C for further analyses.

After blood sampling, birds were sacrificed by cervical dislocation immediately and the pectorals and liver were excised. The pH of the digesta in the jejunum and ceca was subsequently measured with a calibrated digital pH meter with a glass-tipped probe (model IQ120; IQ Scientific Instruments Inc., Carlsbad, CA, USA). Two independent pH readings were taken in each location along the digestive tract. The cecal samples and the content samples of the ileal (Meckel’s diverticulum up to 40 mm above the ileo-cecal junction) digesta were aseptically collected and placed on ice for transportation to the laboratory, where fresh cecal samples were diluted 10-fold by weight in buffered peptone water and homogenized using a stomacher. Viable counts of bacteria in the samples were then determined by plating serial 10-fold dilutions (in 10 g/L peptone solution) onto MacConkey agar, *Lactobacilli* MRS agar, and *Bifidobacterium* agar plates (Beijing Luqiao Technology Limited by Share Ltd, Beijing, China) to verify the *Escherichia coli* (E. coli), *Lactobacillus*, and *Bifidobacterium*, respectively. Agar plates were incubated at 37°C for 36 or 48 h, after which bacterial colonies were counted. Concentration of microflora was finally expressed as log10 colony-forming units per gram of intestinal content.

At the 39th to 42nd day of the experiment, the feed intake was recorded, and all excreta was collected for 4 consecutive days. Excreta were collected from plastic trays placed under the cages 6 times per day (06:00, 9:00, 12:00, 15:00, 18:00 and 21:00 h) and stored at −20°C. Finally, feces from each cage were collected, mixed and weighed. Fecal samples were dried in an oven at 65°C for 48 h. Feed and excreta samples were ground through a 0.45 mm screen and stored for further analysis.

### Chemical analysis

The nutrient contents of feed and excreta samples were analyzed by the methods of the Association of Official Analytical Chemists (AOAC) for DM (Method 930.15), crude protein (CP) (Method 984.13), phosphorus (P) (Method 995.11), and calcium (Ca) (Method 927.02) [19]. The gross energy content of feed and excreta was determined in triplicate 0.6 g samples using an adiabatic bomb calorimeter (IKA Werke, C2000; GMBH, and Co., Staufen, Germany) with benzoic acid as the calibration standard. Copper concentrations of feed, tissue and plasma were analyzed by Flame Atomic Absorption Spectroscopy (Method 999.10 [19]; Shimadzu Scientific Instruments, Kyoto, Japan). The activities of serum ceruloplasmin were determined by using Cu-Zn superoxide dismutase (SOD) typed assay kit (Hydroxylamine method; Nanjing Jiancheng Bioengineering Institute, Nanjing, China). The activities of serum Cu-Zn SOD were determined by using a ceruloplasmin Enzyme Linked Immunosorbent Assay (ELISA) kit (Shenzhen Zike Biotechnology Co., Ltd., Shenzhen, China).

### Statistical analysis

All data were analyzed as a 2×2+1 factorial experiment based on a completely randomized design using the General Linear Model procedure of the SAS software (Statistical Analysis System, Version 9.13, 2002). The effects of the addition of copper source (copper sulfate and cupric citrate) and copper level (50 mg/kg and 100 mg/kg), as well as their interaction (copper source×copper level), on the variables were analyzed using two factor analysis of variance according to the experimental model: Y*_ijk_* = μ+(copper source)*_i_*+(copper level)*_j_*+(copper source×copper level)*_ij_*+e*_ijk_*.

To analyze whether there is a difference between any two groups, Duncan’s new multiple range test was performed. Statistical significance was set at p<0.05.

## RESULTS

### Growth performance

Table 3 presents the impact of copper sulfate and cupric citrate on the growth performance of broilers. The ADFI remained unaffected by copper, irrespective of its source or concentration during any period. However, the ADG was influenced by the copper source during the 1 to 21 day period (p = 0.026) and the overall 1 to 42 day period (p = 0.025), with the cupric citrate groups showing higher values. Furthermore, the F/G was lowest in the CC-100 group.

### Nutrient utilization

Table 4 shows the effect of copper sulfate and cupric citrate on the apparent digestibility of nutrients in broilers. Neither copper sulfate nor cupric citrate affected the apparent digestibility of DM, Ca, or P. However, dietary copper supplementation enhanced the digestibility of energy compared to the control group. Moreover, the groups treated with cupric citrate exhibited higher energy digestibility than those treated with copper sulfate (p = 0.004). The digestibility of CP, on the other hand, was affected by the dietary copper levels (p = 0.046), rather than its sources. Diets with a higher concentration of copper were found to increase the CP digestibility.

### Intestinal microflora

Table 5 presents the impact of copper sulfate and cupric citrate on intestinal microflora of broilers. Copper supplementation significantly reduced the concentration of *E. coli* in the ileum and cecum. This effect was particularly pronounced in the group that received cupric citrate at a higher concentration (p<0.05). A similar trend was observed in the concentration of *Lactobacillus* in the cecum (p<0.05). These findings suggest that cupric citrate, especially at a higher copper dosage of 100 mg/kg, is more effective in diminishing the presence of *Lactobacillus* and *E. coli* in the ileum and cecum.

### Gastrointestinal pH

Table 6 illustrates the effect of copper sulfate and cupric citrate on gastrointestinal pH in broilers. Neither copper sulfate nor cupric citrate influenced the pH in the glandular stomach, muscular stomach, ileum, or cecum. However, dietary copper supplementation led to an increase in the pH in the duodenum (p<0.05) when compared to the control group. In the jejunum, the pH was found to be dosage dependent, with the addition of 100 mg/kg copper (irrespective of the sources) causing a notable increase in the pH in broilers (p = 0.039).

### Antioxidant defences

Table 7 presents the impact of dietary copper sulfate and cupric citrate on antioxidant defense parameters in broilers serum. The copper source influenced ceruloplasmin activities, with CC-100 being the only treatment that significantly increased ceruloplasmin concentration (p = 0.014) compared to the control group. The activity of serum Cu-Zn SOD was also affected by the copper sources, with the highest values observed in the groups treated with cupric citrate (p = 0.025).

## DISCUSSION

Dietary supplementation of chelated copper has been reported to be significantly more effective than adding copper sulfate [8,10,20]. Our results showed that there were no significant differences in ADFI, ADG and F/G of the diets supplemented with cupric citrate and copper sulfate at any stage of the experiment. However, there is a trend that broilers from the CC-50 or CC-100 groups had a higher ADG, indicating cupric citrate promoted the growth of broilers. This can be explained by the increased feed intake in the cupric citrate groups compared to the copper sulfate groups. After being consumed by the birds, copper sulfate encounters moisture in the mouth and the gastrointestinal tract and release free copper ions [21]. Exposure to free copper ions causes damage to the mucous membrane of mouth and gastrointestinal tract [22], leading to reduced feed intake in the copper sulfate-fed birds. Moreover, we also showed that adding 50 mg/kg cupric citrate in the diet improved the growth performance of broilers, which is comparable to broilers with 100 mg/kg dietary copper sulfate. This provides a new idea for adding low-dose cupric citrate instead of high-dose copper sulfate to provide copper for broilers in practical production.

Adding copper to feed can improve nutrient digestion efficiency by increasing the activity of related enzymes. For instance, appropriate copper ions activates pepsin activity and promote protein absorption in pigs [23]. In addition, high dietary copper improves the activities of lipase and phospholipase A in the small intestine, thus improving the absorption of essential fatty acids in weaning pigs [24]. We previously reported that adding copper to mink diet can activate fat digestive enzymes and pepsin, thus improving the digestibility of protein in the diet [25]. Consistent with these findings, our experiment showed a noticeable trend indicating that dietary copper supplementation enhanced the digestibility of energy and CP, with the CC-100 group showing a significant increase in energy utilization compared to the control group. Meanwhile, copper supplementation did not affect the digestibility of calcium and phosphorus. Although not all results reached statistical significance, the observed trends suggest potential benefits of copper supplementation for improving nutrient absorption.

Another factor affecting nutrients digestibility is the pH in the gastrointestinal tract [26]. Jejunum is the main place for nutrient digestion and absorption in broilers, and pH is an essential factor for the activities of digestive enzymes in the jejunum [27]. Thus, it is crucial to maintain pH homeostasis within Jejunum. Under normal conditions, the pH of gastrointestinal tract varies greatly. The stomach has a fairly strong acidity of pH = 1–3. In the intestines, the pH increase from 5 to 6 in the duodenum 6 to 7 in the jejunum and ileum, and 7 to 8 in the cecum [28,29]. Although the exact optimum pH for most enzymes in the broiler digestive tract has not been defined, pH is clearly a major factor influencing nutrient digestibility. Our study demonstrated for the first time that dietary supplementation of 50 mg/kg or 100 mg/kg copper, regardless of its source, slightly increased pH in the jejunum and duodenum but not in the stomach of broilers. Combining with the growth and nutrient utilization performances, our results indicated that a slightly higher pH might be beneficial to the digestion and absorption of substances in the intestines.

Copper is known for its antimicrobial properties. Cupric citrate reduces populations of *E. coli* while increasing *lactobacillus* levels in the cecum, leading to a decrease in both the diarrhea rate and mortality rate in animals [15]. The present study showed that dietary cupric citrate exerts a significant inhibitory effect on *E. coli* in the ileum and cecum of broilers. Notably, the sensitivity of *E. coli* to cupric citrate appears to be dosage dependent. These findings are consistent with a prior research demonstrating copper can significantly reduce bacterial growth and prevent bacterial infections [30]. The mechanisms by which copper eliminates microorganisms are multifaceted and vary across different species. For instance, it is reported that copper inhibits iron-mediated DNA oxidative damage in *E. coli* [31]. In addition, copper has been found to suppress the growth of both wild-type and mutant strains of *E. coli.* The addition of branched-chain amino acids restores this growth, suggesting that copper interferes with their biosynthesis [32]. However, administering a dose of copper that exceeds the required nutritional level may result in reduced food intake and nutrient digestibility. Elevated copper concentrations have been observed to significantly increase the presence of *Dehalobacterium*, *Coprococcus*, and *Spirochaetales* in the rectum, while markedly decreasing the populations of *Salinicoccus*, *Bacillales*, and *Staphylococcus*. This disruption of the microbiota dynamic equilibrium is attributed to the excess copper intake [33]. In light of these findings, the practical application of copper as an antimicrobial agent in animal diets warrants careful consideration to balance its benefits against potential disruptions to the microbiome.

Copper plays a crucial role in animal health as a key component of the antioxidant enzymes ceruloplasmin and Cu-Zn SOD. These enzymes are vital for neutralizing harmful free radicals and maintaining overall health [34]. Ceruloplasmin, in particular, prevents the production of free radicals from iron ions through its ferrous oxidase activity [35]. Similarly, Cu-Zn SOD protects cell membranes by inhibiting superoxide anion free radicals [36]. Numerous studies have demonstrated that a diet high in copper can significantly boost the activities of ceruloplasmin and Cu-Zn SOD in animal serum, contributing to better health outcomes [37,38]. These reports confirm our findings that dietary supplementation with cupric citrate or copper sulfate enhances the activities of antioxidant enzymes in the serum of broilers. This increase in enzyme activity strengthens the animals’ antioxidant defenses and helps maintain redox homeostasis, contributing to their overall health.

## CONCLUSION

Our results demonstrated that adding copper to the diet of broilers generally benefits their growth, nutrient utilization, intestinal microflora, gastrointestinal pH, and antioxidant defenses. Notably, cupric citrate provides superior advantages compared to copper sulfate in these parameters, with effects being dosage-dependent. Our study suggests that a diet supplemented with cupric citrate (100 mg/kg copper) improves broiler’s performance more effectively than copper sulfate, likely due to the superior stability and bioavailability of cupric citrate. Using cupric citrate as a dietary supplement can be a more cost-effective and environmentally friendly option to enhance the health and productivity of broilers.

## Figures and Tables

**Table 1:** Ingredient and chemical composition of basal diet

Item	Starter	Finisher
Ingredient (%)		
Corn	55.2	57.6
Soybean meal (48% CP)	37.0	34.0
Soybean oil	3.0	4
Dicalcium phosphate	2.0	1.4
Ground limestone	0.8	0.9
Salt	0.4	0.4
DL-Methionine	0.3	0.3
L-Lysine	0.3	0.4
Premix^1)^	1	1
Analyzed composition		
Metabolizable energy^2)^ (MJ/kg DM)	12.40	12.76
Dry matter (g/kg)	912.1	903.4
Crude protein (g/kg DM)	220.3	207.8
Contents of animo acid (%)		
Lysine	1.53	1.55
Methionine	0.63	0.62
Methionine+cysteine	0.99	0.95
Contents of mineral elements (mg/kg)		
Calcium	10,016	8,641
Available phosphorus	5,476	4,239
Copper	7.81	7.03

CP, crude protein; DM, dry matter.

1) Provided per kilogram of diet: 11,000 IU vitamin A; 1,800 IU vitamin D_3_; 32 mg vitamin E; 0.5 mg vitamin K_3_; 2.5 mg vitamin B_1_; 8 mg riboflavin; 4.5 mg vitamin B_6_; 26 mg vitamin B_12_; 65 mg niacin; 1.5 mg folic acid; 0.2 mg biotin; and 18 mg pantothenic acid; 60 mg Zn (as ZnSO_4_); 95 mg Mn (as MnO_2_); 65 mg Fe (as FeSO_4_·7H_2_O); 0.7 mg I (as KI); and 0.2 mg Se (as Na_2_SeO_3_·5H_2_O).

2) Calculated values, others were analyzed values based on dry matte samples.

**Table 2:** The final copper content of the diets (mg/kg)

Item	Additive copper does	Actual copper does (Starter)	Actual copper does (Finisher)
Control	0	7.81	7.03
CS-50	50	57.74	57.12
CS-100	100	107.32	107.57
CC-50	50	58.22	57.12
CC-100	100	108.25	107.69

CS, copper sulfate; CC, cupric citrate.

**Table 3:** Effect of copper sulfate and cupric citrate on growth performance of broilers

Item^1)^	Day 1 to 21			Day 22 to 42			Day 1 to 42		
		
	ADG (g/d)	ADFI (g/d)	F/G	ADG (g/d)	ADFI (g/d)	F/G	ADG (g/d)	ADFI (g/d)	F/G
Control	36.40	49.72	1.37	85.47	170.82	2.00	60.93	110.27	1.81
CS-50	36.67	49.33	1.34	86.73	169.55	1.96	61.70	109.44	1.77
CS-100	38.23	50.51	1.32	87.25	171.03	1.96	62.74	110.77	1.77
CC-50	38.15	49.83	1.31	89.34	175.14	1.96	63.75	112.48	1.77
CC-100	38.56	51.32	1.33	89.45	171.70	1.92	64.01	111.51	1.74
SEM	0.51	0.90	0.02	1.40	2.89	0.03	0.78	1.62	0.02
Source mean									
CS	37.45	49.92	1.33	86.99	170.29	1.96	62.22	110.11	1.77
CC	38.36	50.58	1.32	89.40	173.42	1.94	63.88	112.00	1.76
Level mean									
50 mg/kg	37.41	49.58	1.33	88.04	172.35	1.96	62.73	110.96	1.77
100 mg/kg	38.40	50.92	1.33	88.35	171.37	1.94	63.38	111.14	1.76
p-value									
Source	0.026	0.716	0.274	0.103	0.594	0.402	0.025	0.531	0.199
Level	0.090	0.185	0.954	0.838	0.760	0.604	0.454	0.919	0.547
Interaction	0.316	0.880	0.311	0.894	0.443	0.492	0.654	0.519	0.782

Data are means of 12 observations per treatment.

ADG, average daily gain; ADFI, average daily feed intake; F/G, feed gain ratio; CS, copper sulfate; CC, cupric citrate; SEM, standard error of the mean.

1) Comparisons between: Source, CS vs CC; Level, 50 mg/kg copper dosage vs 100 mg/kg copper dosage; Interaction, source and level.

**Table 4:** Effect of copper sulfate and cupric citrate on apparent nutrients digestibility in broilers

Item^1)^	DM (%)	Energy (%)	CP (%)	Ca (%)	P (%)
Control	73.27	75.34^b^	50.99	37.98	26.16
CS-50	73.84	78.22^ab^	51.44	38.45	26.48
CS-100	75.28	79.01^ab^	53.29	38.95	26.50
CC-50	74.66	79.20^ab^	52.61	37.66	26.07
CC-100	75.74	79.91^a^	53.91	38.42	26.95
SEM	0.95	0.86	0.69	0.82	0.67
Source mean					
CS	74.56	78.62^b^	52.37	38.70	26.49
CC	75.20	79.56^a^	53.26	38.04	26.51
Level mean					
50 mg/kg	74.25	78.71	52.03^b^	38.06	26.28
100 mg/kg	75.51	79.46	53.60^a^	38.69	26.73
p-value					
Source	0.337	0.004	0.064	0.711	0.920
Level	0.238	0.432	0.046	0.489	0.546
Interaction	0.864	0.963	0.718	0.886	0.566

Data are means of 12 observations per treatment.

DM, dry matter; CP, crude protein; Ca, calcium; P, phosphorus; CS, copper sulfate; CC, cupric citrate; SEM, standard error of the mean.

1) Comparisons between: Source, CS vs CC; Level, 50 mg/kg copper dosage vs 100 mg/kg copper dosage; Interaction, source and level.

a,b Means in a column without common superscripts differ (p<0.05).

**Table 5:** Effect of copper sulfate and cupric citrate on intestinal microflora in broilers

Item^1)^	Ileum (log_10_ cfu/g)		Cecum (log_10_ cfu/g)	
	
	*Lactobacillus* spp.	*Escherichia coli*	*Lactobacillus* spp.	*Escherichia coli*
Control	7.35	5.39^a^	8.40^a^	7.31^a^
CS-50	7.05	4.72^ab^	7.94^ab^	6.86^a^
CS-100	6.43	4.36^bc^	7.27^bc^	5.86^bc^
CC-50	6.93	4.56^bc^	7.28^bc^	6.09^b^
CC-100	6.55	3.98^c^	6.83^c^	5.36^c^
SEM	0.23	0.16	0.20	0.16
Source mean				
CS	6.74	4.54^a^	7.61^a^	6.36^a^
CC	6.74	4.27^b^	7.06^b^	5.73^b^
Level mean				
50 mg/kg	6.99	4.64^a^	7.61^a^	6.48^a^
100 mg/kg	6.49	4.17^b^	7.05^b^	5.61^b^
p-value				
Source	0.110	0.000	0.000	0.000
Level	0.055	0.013	0.016	0.000
Interaction	0.638	0.527	0.626	0.433

Data are means of 12 observations per treatment.

CS, copper sulfate; CC, cupric citrate; SEM, standard error of the mean.

1) Comparisons between: Source, CS vs CC; Level, 50 mg/kg copper dosage vs 100 mg/kg copper dosage; Interaction, source and level.

a–c Means in a column without common superscripts differ (p<0.05).

**Table 6:** Effect of copper sulfate and cupric citrate on gastrointestinal pH in broilers

Item^1)^	Glandular stomach	Muscular stomach	Duodenum	Jejunum	Ileum	Cecum
Control	2.96	3.78	5.86^b^	6.03	6.00	7.1
CS-50	2.92	3.75	6.02^ab^	6.06	6.10	7.08
CS-100	2.92	3.73	6.19^a^	6.18	5.92	6.93
CC-50	2.99	3.82	6.09^ab^	6.13	6.09	7.13
CC-100	2.92	3.78	6.25^a^	6.28	5.94	6.97
SEM	0.04	0.05	0.05	0.06	0.09	0.09
Source mean						
CS	2.92	3.74	6.11^b^	6.12	6.01	7.01
CC	2.96	3.80	6.17^a^	6.21	6.02	7.05
Level mean						
50 mg/kg	2.96	3.79	6.06^b^	6.10^b^	6.10	7.11
100 mg/kg	2.92	3.76	6.22^a^	6.23^a^	5.93	6.95
p-value						
Source	0.700	0.524	0.001	0.077	0.984	0.711
Level	0.404	0.650	0.011	0.039	0.094	0.115
Interaction	0.497	0.855	0.921	0.828	0.926	0.916

Data are means of 12 observations per treatment.

CS, copper sulfate; CC, cupric citrate; SEM, standard error of the means.

1) Comparisons between: Source, CS vs CC; Level, 50 mg/kg copper dosage vs 100 mg/kg copper dosage; Interaction, source and level.

a,b Means in a column without common superscripts differ (p<0.05).

**Table 7:** Effect of copper sulfate and cupric citrate on antioxidant defense in broilers

Item^1)^	Ceruloplasmin (U/L)	Cu/Zn-SOD (U/mL)
Control	1.93^b^	90.75
CS-50	2.00^ab^	95.98
CS-100	2.07^ab^	98.63
CC-50	2.05^ab^	103.29
CC-100	2.09^a^	106.69
SEM	0.03	3.77
Source mean		
CS	2.04^b^	97.31^b^
CC	2.07^a^	104.99^a^
Level mean		
50 mg/kg	2.03	99.64
100 mg/kg	2.08	102.66
p-value		
Source	0.014	0.025
Level	0.113	0.471
Interaction	0.580	0.927

Data are means of 12 observations per treatment.

Cu/Zn SOD, Cu-Zn superoxide dismutase; CS, copper sulfate; CC, cupric citrate; SEM, standard error of the mean.

1) Comparisons between: Source, CS vs CC; Level, 50 mg/kg copper dosage vs 100 mg/kg copper dosage; Interaction, source and level.

a,b Means in a column without common superscripts differ (p<0.05).
